# A unique case of pulmonary minimally invasive mucinous adenocarcinoma arising from atypical goblet cell hyperplasia in the bronchial epithelium of a 9-year-old girl

**DOI:** 10.1186/s12887-025-05683-9

**Published:** 2025-04-28

**Authors:** Ping Zhou, Cuomu Zhaxi, Lili Jiang

**Affiliations:** 1 Department of Pathology, West China Hospital, Sichuan University, Chengdu, Sichuan, China; 2 Department of Pathology, West China Tibet Hospital, Sichuan University, Tibet, China

**Keywords:** Pediatric oncology, Pulmonary adenocarcinoma, Minimally invasive mucinous adenocarcinoma, VATS

## Abstract

**Background:**

Pulmonary mucinous adenocarcinoma without congenital pulmonary airway malformation (CPAM) is extremely rare in pediatric patients. Here, we report a unique case of minimally invasive mucinous adenocarcinoma without CPAM in a child and provide a comprehensive review of the clinical, radiographic, and histopathological characteristics of the published literature.

**Case presentation:**

A 9-year-old girl presented with persistent cough and sputum production, raising suspicion of respiratory infection. Chest computed tomography (CT) revealed a solid nodule measuring 1.9 cm × 1.6 cm in the right lower lobe. Prenatal ultrasonography revealed no congenital lung abnormality. The patient subsequently underwent video-assisted thoracoscopic surgery (VATS) without postoperative complications. Histologically, a focal area demonstrated marked atypical goblet cell hyperplasia in the bronchial epithelium, which abruptly transitioned to mucinous adenocarcinoma, predominantly characterized by a lepidic growth pattern and extensive extracellular mucin accumulation. Pathological examination confirmed pulmonary minimally invasive mucinous adenocarcinoma, staged as pT1miN0M0. Next-generation sequencing (NGS) identified the *KRAS G12D* mutation. The patient remained well 11 months after resection and did not require additional treatment.

**Conclusions:**

We demonstrated a novel stepwise progression originating from atypical goblet cell hyperplasia in the bronchial epithelium, rather than from the CPAM, in a pediatric patient. *KRAS* mutations may play a critical role in the development of pulmonary mucinous adenocarcinoma in pediatric patients.

## Introduction

Malignant pulmonary neoplasms are rare in pediatric patients [1]. The rarity and nonspecific clinical manifestations pose challenges to accurate recognition and management. Recurrent pneumonia with nodular lesions on chest radiographs that fail to respond to medical treatment should raise suspicion of pulmonary malignancy. Primary pulmonary malignancies may present as ground‒glass opacities (GGOs), solid masses, or mixed nodules on chest computed tomography (CT). Early clinical suspicion of malignant pediatric pulmonary neoplasms can lead to timely surgical interventions, such as video-assisted thoracoscopic surgery (VATS). A definitive diagnosis is established on the basis of histopathological findings.

Pulmonary adenocarcinoma is extremely rare in pediatric patients, and pulmonary mucinous adenocarcinoma is frequently associated with congenital pulmonary airway malformations (CPAM) [2, 3]. Mucinous cell clusters and/or atypical goblet cell hyperplasia in CPAM are considered precursor lesions for pulmonary mucinous adenocarcinoma during childhood [4–6], and *KRAS* mutations may indicate potential malignant behavior [2, 7–10]. It is challenging to suspect primary mucinous adenocarcinoma in pediatric patients without congenital cystic lesions. Herein, we present a unique case of pulmonary minimally invasive mucinous adenocarcinoma harboring a *KRAS* mutation in a 9-year-old girl without CPAM and provide a literature review of its clinical, radiographic, and histopathological features.

## Case presentation

**Fig. 1:**
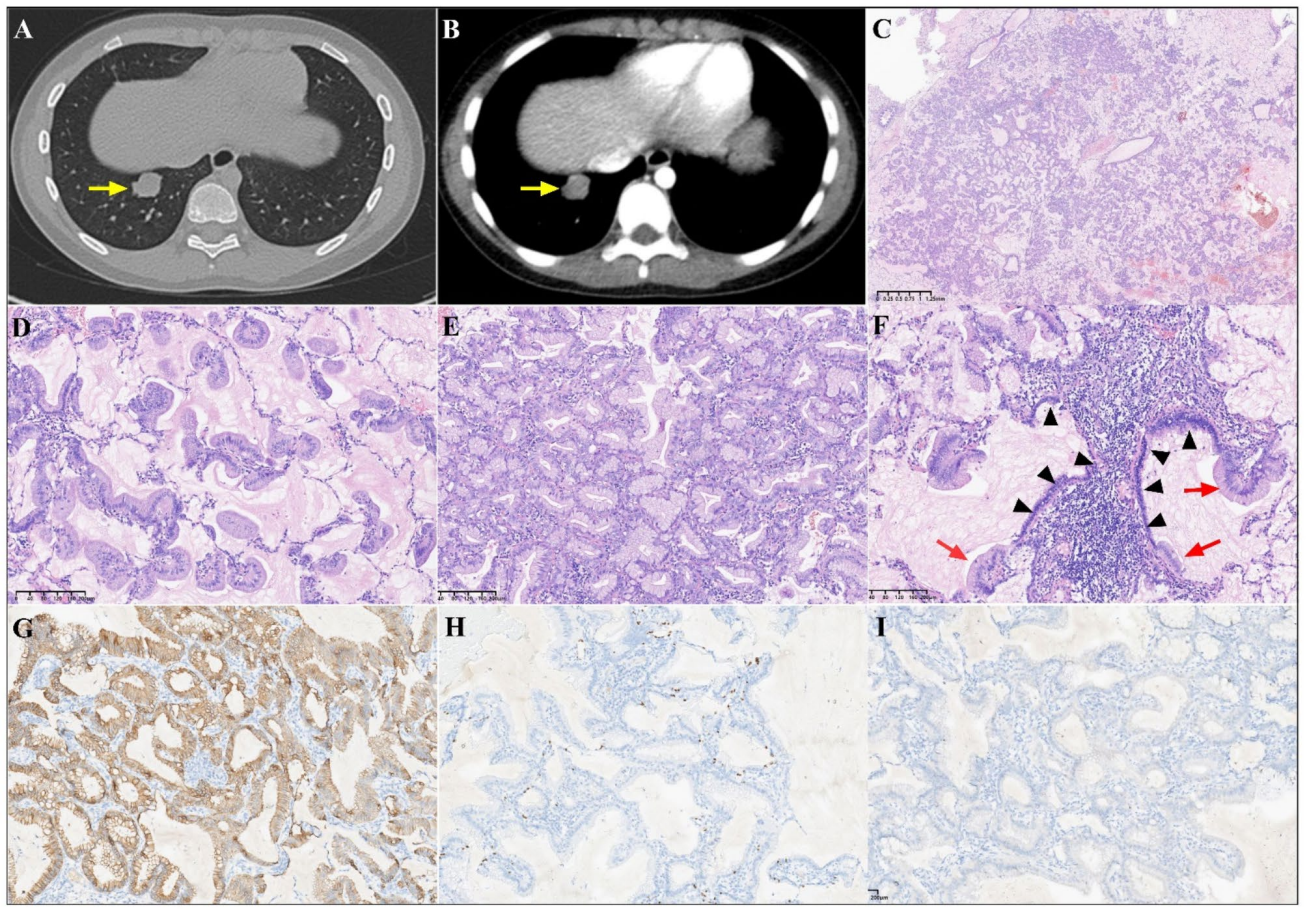
Radiological, histopathological, and immunohistochemical features of pulmonary minimally invasive mucinous adenocarcinoma. Chest CT revealed a solid nodule measuring 1.9 cmx1.6 cm in the posterior basal segment of the right lower lobe (**A**, arrow) with irregular margins and slight enhancement (**B**, arrow). The resected tumor specimen showed a predominantly lepidic pattern with mucous secretion under low-power view (**C**). Middle-power magnification revealed tumor cells with abundant apical cytoplasmic mucin and small, basally oriented nuclei growing along the surface of the preserved alveolar walls (**D**). The partial tumor exhibited an invasive pattern of infiltrative glands lined by tumor cells containing abundant intracytoplasmic mucin (**E**). The focal area exhibited atypical goblet cell hyperplasia (**F**, arrows**)** in the bronchial epithelium (**F**, triangles). (Magnification x100). Immunohistochemical analysis revealed positive staining for CK7 (**G**) and negative staining for TTF-1 (**H**). CK5/6 staining revealed no evidence of basal cell layer (**I)**. (Magnification ×100)

A 9-year-old girl with persistent cough and sputum production lasting for three weeks was admitted to a local hospital. Despite antibiotic treatment for the suspected respiratory infection, the symptoms did not improve. Chest CT revealed a nodule in the right lower lobe, leading to the patient’s admission to our hospital for further treatment. Contrast-enhanced chest CT revealed a solid nodule measuring 1.9 cm × 1.6 cm in the posterior basal segment of the right lower lobe (Fig. 1A) with irregular margins and slight enhancement (Fig. 1B). The patient successfully underwent VATS of the right lower lobe without postoperative complications.

Histopathological examination of the resected tumor revealed a predominantly lepidic growth pattern with mucin secretion under low-power view (Fig. 1C). Middle-power magnification revealed tumor cells with abundant apical cytoplasmic mucin and small, basally oriented nuclei (Fig. 1D). The partial tumor exhibited an invasive pattern of infiltrative glands lined by tumor cells containing abundant intracytoplasmic mucin, measuring less than 5 mm in size (Fig. 1E). Notably, the focal area exhibited atypical goblet cell hyperplasia (Fig. 1F, arrows**)** in the bronchial epithelium (Fig. 1F, triangles). The resection margin and lymph nodes were negative. The immunohistochemical staining results were as follows: CK7 was positive (Fig. 1G), TTF-1 was negative (Fig. 1H), and CK5/6 staining revealed no evidence of a basal cell layer (Fig. 1I). Next-generation sequencing (NGS) analysis of 1021 cancer-related genes by Geneplus Technology identified the *KRAS G12D* mutation. No mutations were detected in *EGFR*, *ALK*, *ROS-1*, or *TP53* genes.

The final pathological diagnosis was pulmonary minimally invasive mucinous adenocarcinoma (MIA, mucinous type), staged as pT1miN0M0. The patient remained well 11 months after resection and did not require additional treatment.

## Discussion

Malignant pediatric pulmonary neoplasms are rare, with an age-adjusted incidence of 0.049 per 100,000 individuals and a median age at diagnosis of 16 years. Pulmonary adenocarcinoma is extremely rare in pediatric patients, accounting for only 6% of all malignant pulmonary neoplasms [1]. The clinical manifestations of malignant pediatric pulmonary neoplasms are nonspecific and include persistent cough, recurrent pneumonia, hemoptysis, tachypnea, dyspnea, or even asymptomatic presentations. Shahkar L. et al. reported a rare case of pulmonary adenocarcinoma in an 8-year-old patient with persistent respiratory manifestations, including a non-purulent cough, dyspnea, and hemoptysis [11]. Primary pulmonary malignancies may manifest as incidentally detected small lung nodules on chest CT scans and predominantly present as GGOs. Lei et al. reported the case of an 11-year-old child who underwent VATS for bilateral wedge resection to diagnose minimally non-mucinous invasive adenocarcinoma presenting as two GGOs [12]. In a large cohort case series reported by Wu et al., GGOs were detected in 12 adolescents with early-stage pulmonary adenocarcinoma, including ten patients with minimally invasive adenocarcinoma (MIA) and two patients with invasive adenocarcinoma (IAC) [13]. Pulmonary mucinous adenocarcinoma typically manifests as a solid mass or nodule on chest CT. Differential diagnoses include congenital lung abnormalities such as pulmonary sequestration (PS) and CPAM, tuberculosis, hamartoma, pleuropulmonary blastoma, mucoepidermoid carcinoma, pulmonary inflammatory myofibroblastic tumor, fetal lung interstitial tumor (FLIT), neuroendocrine tumors, and sarcoma. In this report, we describe a case of pulmonary minimally invasive mucinous adenocarcinoma that presented as a solid nodule on chest CT and was associated with persistent cough and sputum production. Symptom onset occurred approximately one month before surgical treatment.

Pulmonary mucinous adenocarcinoma is most commonly associated with CPAM [2–4]. CPAM is the most common congenital lung anomaly, with type 1 CPAM being the predominant subtype [14]. Mucinous cell clusters and/or atypical goblet cell hyperplasia in CPAM are considered potential precursors of pulmonary adenocarcinoma in childhood [4–6, 10]. They are typically diagnosed as mucinous adenocarcinoma in situ (AIS), minimally invasive mucinous adenocarcinoma (MIA), or invasive mucinous adenocarcinoma (IMA), depending on the size and degree of invasion. IMAs arising from CPAMs mainly exhibit an indolent clinical course; however, recurrence or metastasis may occur when these IMAs are inadequately resected [10]. Chang, W. C. et al. reported the largest series of 37 cases of mucinous adenocarcinomas arising from CPAM; 22 patients were under 18 years old, with tumor stages distributed as follows: stage 0 (pTis; 15/22), stage I (pT1 and pT2a; 6/22), and stage II (pT2b; 1/22) [2]. In addition to its association with CPAM, pulmonary adenocarcinomas have been reported in pediatric patients who have undergone prolonged treatment for other malignancies. Three cases of lung adenocarcinoma were identified in children aged 8–17 years during the progression of bone osteosarcoma or Ewing sarcoma [15]. A 13-year-old boy was diagnosed with primary pulmonary adenocarcinoma three years after the diagnosis of osteosarcoma [16]. Additionally, a 9-year-old child had a synchronous occurrence of minimally invasive adenocarcinoma and an inflammatory myofibroblastic tumor in the lungs [17].

However, there are few studies on mucinous adenocarcinoma without CPAM [18, 19]. Achuta K et al. reported a case of pediatric primary lung adenocarcinoma in a 17-year-old boy without CPAM; the histological features revealed non-mucinous adenocarcinoma [18]. Turut et al. reported a case of primary pulmonary mucinous adenocarcinoma in a 15-year-old boy. The tumor cells had eosinophilic cytoplasm and round or oval nuclei and formed adenoid and cribriform patterns, which represented non-mucinous adenocarcinoma with extensive mucin production filling the alveolar spaces [19]. Three cases of pulmonary adenocarcinoma aged 8–17 years without CPAM did not provide information regarding the histological subtype of adenocarcinoma and did not include any histological images [11, 20, 21]. In the present case, we provide comprehensive clinicopathological characteristics, highlighting atypical goblet cell hyperplasia in the bronchial epithelium as histological evidence of premalignant changes, which are distinct from those typically associated with CPAM.

Pulmonary mucinous adenocarcinoma has been reported to predominantly harbor *KRAS* oncogene driver mutations in pediatric patients, particularly in association with CPAM [2–4]. Chang et al. reported that 90% (9/10) of mucinous adenocarcinomas arising in the CPAM harbored *KRAS* mutations, including five cases with the *KRAS G12D* mutation and four cases with the *KRAS G12V* mutation [2]. A 10­-day-old male neonate presented with type 2 CPAM in the left lower lobe complicated by multifocal mucinous adenocarcinoma harboring the *KRAS G12D* mutation [3]. Koopman et al. reported a 6-week-old girl with type 1 CPAM; histopathological analysis of the resected tissue revealed non-mucinous papillary and mucinous cluster areas, both harboring the *KRAS G12D* mutation, which is considered potentially premalignant [22]. Notably, mucinous adenocarcinoma with intrapulmonary metastasis harboring concurrent *KRAS* and *GNAS* mutations was identified in a 14-year-old boy with type 1 CPAM [23], suggesting that *GNAS* mutations may play a critical role in tumor progression and metastasis. The present case of pulmonary minimally invasive mucinous adenocarcinoma without coexisting CPAM harbored the *KRAS* G12D mutation, which is consistent with previous reports. Therefore, *KRAS* mutations play an essential role in the development of pulmonary mucinous adenocarcinomas in pediatric patients.

Managing pulmonary adenocarcinoma in pediatric patients remains challenging and may be influenced by factors such as the tumor-node-metastasis (TNM) stage and patient performance status. Surgical intervention has been shown to significantly improve the 10-year survival to 75% in pediatric patients with malignant pulmonary neoplasms [1]. A meta-analysis of 903 children who underwent thoracoscopic lobectomy and 1192 who underwent thoracotomy demonstrated that thoracoscopic lobectomy resulted in a lower incidence of overall and respiratory complications than thoracotomy in children with congenital lung lesions [24]. VATS lobectomy is recognized as a safe and effective procedure in children [25]; it can be used to manage specific benign pulmonary conditions and address T1-T2 N0 M0 bronchogenic carcinomas in adult patients [26]. Among 12 patients aged 13–20 years with incidentally detected GGOs (mean tumor size 0.93 ± 0.25 cm on chest CT), all underwent VATS lobectomy, and no recurrence was observed during a median follow-up of 12.5 months [13]. Therefore, VATS lobectomy may be a reliable option for pediatric patients with early-stage pulmonary cancer. The present patient successfully underwent VATS lobectomy and remained well 11 months after resection without requiring any additional treatment.

Owing to their rarity and nonspecific clinical manifestations, some pulmonary adenocarcinomas are diagnosed at an advanced stage, and few cases of metastatic primary pulmonary adenocarcinoma with intraluminal lesions obstructing the left main bronchus [20] or pulmonary non-mucinous adenocarcinoma with brain metastasis have been reported [27]. Some patients may require comprehensive treatment, including radiation therapy [20], chemotherapy [27], and targeted therapy [21]. Megaro et al. reported the case of a 17-year-old male with metastatic ALK-positive adenocarcinoma who was treated with crizotinib and achieved a prolonged response (PFS: 33 months) [21]. Chemotherapy combined with immunotherapy is the preferred first-line treatment for adults with advanced *KRAS*-mutated non-small cell lung cancer (NSCLC)[28]. Among adult patients with *KRAS*-mutated NSCLC, 15% (354/2327) harbor the *KRAS G12D* mutation [29]. Compared with non-*KRAS G12D*,* KRAS G12D* has been reported to be associated with lower PD-L1 expression, a lower tumor mutation burden (TMB), increased immunosuppression, and enhanced resistance to immune checkpoint inhibitors (ICIs) [29, 30]. Liu et al. demonstrated the better efficacy of chemoimmunotherapy than ICI monotherapy in patients with *KRAS* G12D-mutant NSCLC [30]. Therefore, *KRAS* mutations are closely linked to prognosis and treatment response. However, management and treatment options for pediatric lung cancer patients harboring *KRAS* mutations remain limited. Further investigations of effective therapeutic strategies are warranted.

## Conclusions

We present a unique case of pulmonary minimally invasive mucinous adenocarcinoma with *KRAS* G12D mutation in a pediatric patient who underwent VATS. The tumor originated from atypical goblet cell hyperplasia in the bronchial epithelium, rather than from the CPAM. Both atypical goblet cell hyperplasia in the bronchial epithelium and mucinous cell clusters in CPAM may be precursors of pulmonary mucinous adenocarcinoma in pediatric patients.

## Data Availability

The datasets used in the current study are available from the corresponding author upon reasonable request.

## Abbreviations

MIA: Minimally invasive adenocarcinoma
CPAM: Congenital pulmonary airway malformation
VATS: Video-assisted thoracoscopic surgery
CT: Computed tomography
NGS: Next-generation sequencing
